# Accountability for Reasonableness as a Framework for the Promotion of Fair and Equitable Research

**DOI:** 10.1002/hast.4931

**Published:** 2024-12-21

**Authors:** Charles Dupras, Marie‐Pierre Dubé, Simon Gravel, Hazar Haidar

**Keywords:** accountability for reasonableness, A4R, genomic research, diversity, exclusion, procedural justice, equity and fairness, research ethics

## Abstract

Despite increased efforts to ensure diversity in genomic research, the exclusion of minority groups from data analyses and publications remains a critical issue. This paper addresses the ethical implications of these exclusions and proposes *accountability for reasonableness* (*A4R*) as a framework to promote fairness and equity in research. Originally conceived by Norman Daniels and James Sabin to guide resource allocation in the context of health policy, A4R emphasizes publicity, relevance of reasons, enforcement, and revision as essential for legitimacy and trust in the decision‐making process. The authors argue that A4R is also relevant to resource allocation in research and that, if adequately informed and incentivized by funding agencies, institutional review boards, and scientific journals, researchers are well‐positioned to assess data‐selection justifications. The A4R framework provides a promising foundation for fostering accountability in genomics and other fields, including artificial intelligence, where lack of diversity and pervasive biases threaten equitable benefit sharing.

Improving fairness and equity within genomic research, as well as in the broader fields of science and technology, requires an ongoing examination of systematic exclusions of certain groups of people from research participation and the resultant impact on these groups. Over the last decades, significant efforts have been made to establish more diverse and representative biobanks and databases for genomic research.^1^ This effort faces many challenges, however. One involves the ambiguity surrounding what is meant by “diversity.”^2^ Does it encompass biological, social, cultural, and behavioral traits? How exactly should diversity within biobanks and databases be characterized? What is the precise terminology that should be used to do so? How can we distinguish fair from unfair representation of distinct groups and from within which population—at local, national, or international levels? Another challenge is that some individuals and populations are understandably hesitant to participate in genomic research, often due to historic trauma related to colonial research involving their communities or concerns related to the political climate in their countries.^3^ This further complicates the inclusion of all global citizens in collaborative transnational genomic research. In addition, the underrepresentation of certain groups in academia and research teams, compounded by disparities in funding, hinders studies on topics of special relevance to these groups.

Fortunately, many scholars and stakeholders are working hard to address these pressing issues collaboratively, with efforts to increase data collections, harmonize data storage protocols, and facilitate access to more diverse and representative research data. However, two equally important problems arise during the research process: the systematic exclusion of data from minority groups during data analysis, even when such data are available, and the selective reporting of research results in peer‐reviewed publications, where only results based on majority data are presented.

These practices are ethically questionable, especially when substantiated justifications are not presented. In 2020, we showed that, out of fifty‐eight published genome‐wide association studies (GWAS) in which data from the U.K. Biobank or the U.S. Health and Retirement Study was used, forty‐five did not include data from minority groups.^4^ Among these forty‐five publications, thirty‐one provided no justification for excluding minority groups. In the remaining fourteen, the reasons for exclusion included concerns about confounding factors, lower statistical power for minority groups’ data, the time and resources that might be necessary for multiple analyses, and apprehension about potential delays in the peer‐review process when the analyzed datasets are heterogeneous. Consequently, GWAS findings continue to be skewed in favor of majority groups, limiting access to the advantages of genomic technologies for historically marginalized communities because of uncertain benefits and risks if the technologies are used for them.^5^

To address the unwarranted exclusion of minority groups from research, we briefly introduced the idea of importing the accountability for reasonableness (A4R) framework by Norman Daniels and James Sabin from the field of health policy ethics.^6^ Originally conceived as a procedural justice model for ethical decision‐making in health care resource allocation, the A4R framework emphasizes the significance of transparency in decision‐making, the need for providing justifications that are relevant to the issue in question, and the importance of providing an opportunity for all stakeholders to participate in the decision‐making process.^7^ In this essay, we expand on this idea by further exploring the applicability of the A4R framework to promote fairness and accountability of data selection practices. We consider the justification of inclusion or exclusion of data from minority groups throughout the research process, including during data selection, data analysis, and reporting. We discuss how each of the framework's four conditions (publicity, relevance of reasons, enforcement, and revision) can be operationalized to address equity and fairness in genomic research and science and technology more broadly. Our main objective is to raise awareness within the research ethics and integrity community of a framework that has thrived for over two decades in health policy ethics and may prove valuable in enhancing fairness in genomics research in the future.

We aim to address three important gaps. First, there is no systematic method to evaluate data selection practices used by researchers at four stages of the research process. These stages include grant applications’ evaluation by research funding organizations, approval of research projects by institutional review boards (IRBs), approval by data access committees (DACs) responsible for overseeing access to datasets, and peer review assessment of manuscripts for publication in scientific journals. The A4R framework may be useful to guide the implementation of checkpoints for critical assessment of the exclusion of minority groups from research.

Second, our proposal aims to provide a procedural justice framework that actively engages the research community in identifying ethical issues in ongoing data selection practices and result reporting. Given the complexity of the research topics, advanced scientific expertise in the topics of the manuscripts under review for publication is necessary for distinguishing well‐justified data selection and, thus, fair exclusion practices from those that are not. Third, there is a need for stronger incentives to motivate researchers to provide relevant justifications for data exclusion and inclusion. We expect that the prospect of facing more explicit justification requirements on the part of funding agencies, IRBs, DACs, and publishers will serve as such an incentive to researchers. Additionally, the establishment of clearer guidelines will likely facilitate the approbation by researchers of well‐justified data analysis practices.

Below, we describe Daniels and Sabin's A4R framework and provide examples of its implementation in the health care sector over the past twenty‐five years. We highlight the framework's strengths and limitations, drawing on feedback from advocates and critics in the literature, and expand on why and how the framework appears transferable to the research context. We propose that transparency can be achieved when researchers clearly explain and justify data exclusion decisions as part of the funding application, ethics review, data access, and publication processes. We examine some examples of potentially relevant and irrelevant reasons for exclusion of data from minority groups in different contexts. We also argue that enforcing the assessment of relevance necessitates the systematic involvement of funding agencies, IRBs, DACs, publishers, and participant groups that have historically been excluded or are at higher risk of being excluded from research. We conclude by highlighting the importance of open dialogue and constructive deliberation among all stakeholders involved to explore what constitutes fair and unfair data exclusion and the circumstances that determine fairness.

## Accountability for Reasonableness

Daniels and Sabin proposed the A4R framework in their 1997 article “Limits to Health Care: Fair Procedures, Democratic Deliberation, and the Legitimacy Problem for Insurers”^8^ as a procedural method for reconciling fair and equitable health care resource allocation with the practical limitations faced by health care systems. The framework acknowledges the existence of difficult choices and trade‐offs in resource allocation while emphasizing procedural fairness. The initial framework established four essential conditions that must be met to ensure that such choices are fair and equitable: relevance, publicity, appeals, and enforcement.^9^

The relevance condition necessitates that decision‐makers provide justifications for resource‐allocation decisions based on evidence, values, and principles that a fair‐minded person would most likely be willing to consider as legitimate reasons. These justifications should be reasonable and open to critical examination. The publicity condition highlights the importance of transparency in decision‐making, including the disclosure of criteria, principles, and evidence used in the process. By promoting transparency, the framework aims to build trust and foster accountability among stakeholders. The appeals condition acknowledges the complexity of health care allocation decisions and emphasizes the need for ongoing evaluation and refinement. It encourages the establishment of mechanisms through which affected parties can voice their concerns and contribute to improving decisions over time. The enforcement condition underscores the significance of consistently applying agreed‐upon principles and processes for resource allocation. This condition emphasizes the need for oversight and mechanisms to hold decision‐makers accountable for adhering to principles of fairness and reasonableness, thereby reducing the risk of arbitrary or discriminatory practices.^10^

The A4R framework garnered widespread attention in health care, and it has been adopted in diverse contexts in health policy decision‐making, such as guiding health technology assessment, priority setting regarding the public coverage of health care interventions, and the fair allocation of scarce medical resources,^11^ as in the context of organ transplantation.^12^ In such cases, the A4R framework has been found valuable to guide decision‐making on how limited medical interventions should be distributed within society and what criteria should guide these decisions. The primary objective of the framework is to ensure fairness, accountability, and transparency in the allocation of resources and the decision‐making processes surrounding health care interventions.

The A4R framework's relevance extends beyond its traditional applications, however. It can be effectively applied, we believe, in the research context, and particularly in guiding priority setting for scientific studies and addressing the challenges posed by limited research resources. In the realm of scientific research, there is a constant need to make informed decisions about how to allocate funds to support various aspects or costs of the research, such as laboratory equipment, specialized tests, or personnel salaries. The process of allocating limited research resources also includes decisions made by researchers regarding which populations should be considered for study, the size and composition of the samples targeted at the recruitment stage, and the selection of datasets for analysis. These decisions can become problematic when they have the effect of excluding already‐marginalized groups from research for reasons that a fair‐minded person would not consider legitimate. Consequently, research choices, even for researchers with clear equity goals in mind, can pose challenges when conflicting with other personal or research objectives, potentially affecting the overall equity of the study. Therefore, and as an attempt to provide a procedural justice framework to guide these decisions and their ethical assessment by third parties, we propose the implementation of the A4R framework in research.

## Promoting Fair and Equitable Research

When researchers undertake a research study involving human participants, their data, or some of their biological materials, the research project typically undergoes multiple stages of evaluation to ensure its scientific quality, ethical integrity, and the realization of its methods and goals. These evaluation steps usually include a grant review application and evaluation process, research ethics review, and a final peer review process prior to the publication of the research results. At the grant review application stage, researchers seek funding from agencies or institutions. This phase entails, in principle, a thorough and comprehensive evaluation of the research proposal by experts in the field to gauge the project's significance, feasibility, and potential impact. Prior to commencing the funded research, researchers are required to seek approval from IRBs or DACs. These bodies play a crucial role in safeguarding the well‐being and privacy of human participants by evaluating the research design, informed consent process, data collection procedures, and potential risks to participants. Upon completing the research study, researchers typically prepare a manuscript that includes research findings for publication. At this stage, the manuscript undergoes a peer review process by experts in the field. Peer reviewers scrutinize the research methodology, data analysis, results, and conclusions to ensure the study's validity, originality, and contribution to the scientific community. Feedback from the peer review process contributes to improving the manuscript's quality and credibility before publication.

Questions about the fairness of study design can arise at various stages throughout the research process. Therefore, it is essential to implement strategies at all stages to identify and address potentially problematic selection practices. Below, we offer examples of relevant questions that can be addressed by applying the A4R framework to the context of genomics research, as well as in other research contexts. We illustrate how the framework can be used as an analytical grid for developing processes that aim to evaluate the relevance of reasons for exclusion presented by researchers in their funding applications, protocol submissions for IRB or DAC approval, and manuscript submissions for publication. The questions we highlight can be considered by researchers, funders, and members of IRBs or DACs when assessing the suitability of a research proposal for funding, ethics approval, or data access authorization or by editors and peer reviewers when assessing the resulting manuscript from the research proposal.

***Publicity***. At the four stages of research, the review process provides an opportunity to assess the explanations given by researchers for excluding certain datasets from analysis. To facilitate this evaluation, we could establish and implement policies mandating that researchers maintain transparency regarding the facts and reasons behind their decision to include some data in their analysis and exclude other data. As shown previously, such transparency is often lacking, leaving the reviewers and, once a manuscript is published, readers unable to comprehend and concur with these choices. To uphold the principle of transparency, applicants and authors might be invited and encouraged to answer questions such as the following:

- Are there numerous potentially useful databases related to my research topic, and have I selected one among various available options?
- Are there any obstacles to accessing some of the existing databases in your field?
- How well are the selected datasets representative of the global population?

For instance, in genome‐wide association studies (GWAS), it might be advisable to incorporate datasets from multiple sources (for example, All of Us, Human Heredity and Health in Africa,^13^ Qatar Biobank,^14^ South Asia Biobank,^15^ and UK Biobank,^16^ among others) to more comprehensively account for global diversity. Researchers should also consider the level of data diversity and the availability of information related to diversity (such as metadata categories) within the chosen databases. Some circumstances, such as when researchers are piloting new methods and have access to or are familiar with specific databases, may also need to be disclosed and taken into account to evaluate the legitimacy of selectively favoring specific databases or datasets. Mere convenience may be insufficient.

***Relevance of reasons***. Researchers should demonstrate transparency not only with respect to their data selection practices but also, more specifically, concerning the reasons behind decisions to exclude participant groups or datasets from their study. Providing a rationale for the selection of participant groups and cohorts is crucial and should be a recurring process at various stages of research development, including funding approval, review by institutional or ethical review boards or DACs, and manuscript preparation for peer review. This may involve the need for a homogenous sample, either to pilot a new methodological or analytical approach or because selecting a particular approach is considered the only viable way to yield valid and significant scientific results. It is crucial to emphasize that these examples should not be viewed as either necessary or sufficient conditions for justifying exclusion; their relevance may depend on the context. Before submitting their protocols or manuscripts for review, researchers should reflect on at least three important questions:

- What are the scientific justifications for my decision to exclude specific data?
- What are the ethical grounds for my decision to exclude particular data?
- Have practical or technical challenges influenced my decision to exclude specific data?

Satisfactorily addressing any one of these three questions can sometimes suffice to justify exclusion on reasonable grounds. For instance, exclusion might be justified if conducting data analyses using heterogeneous samples would not yield valuable scientific results and increasing diversity is therefore expected to reduce the study's potential societal benefits. However, researchers should also explore the potential use of alternative analytical methods that can circumvent these limitations. Ethical grounds for exclusion could arise when a significant number of participants from a specific population in the biobank or database did not consent to a particular type of analysis or research topic, leaving the researchers with no other choice but to perform data analysis on a subpopulation. Exclusion might occur, for example, out of respect for Indigenous data sovereignty concerning a specific study that was not approved by one or more of the relevant communities. Practical or technical challenges might emerge if the methodological or analytical tools employed in a study (such as one relying on population‐specific reference data) lack prior validations for use in a specific subpopulation. While these justifications do not necessarily make the situation equitable or ideal, they may be considered legitimate grounds for data selection within a particular context. Other acceptable reasons for exclusion may exist. With the guidance of the people in charge of the various stages of research (such as IRB administrators and editors), reviewers with sufficient expertise in the research are best positioned to evaluate the relevance of the reasons provided by authors. This does not negate the possible need to identify criteria for assessing the relevance of these reasons, but such criteria should acknowledge the particular circumstances of each study. Thus, the A4R framework requires that a certain level of trust be placed in the ability of reviewers to make reasonable and explicable judgment calls when necessary.

***Enforcement***. The principle of enforcement is crucial for ensuring adoption of and compliance with the A4R framework's other three principles. The review processes at the four stages of research identified above are ideally suited for this task, as they already serve as gateways for the research. Therefore, incorporating assessments of diversity considerations into the evaluations and regarding participant data selection practices as integral to these evaluations would not necessitate a significant process overhaul. The main reason we believe that the external review process is the ideal for this assessment is that it can serve as a strong incentive for researchers to ensure that, when they exclude data from specific groups, they do so for legitimate and justifiable reasons, which fair‐minded persons with sufficient expertise in their field would acknowledge. This requires researchers to reflect on their protocol, starting from the early stages of study design and funding, through recruitment and data analyses, and culminating in the reporting of results in their papers. In the end, what matters is that the enforcers be given both the opportunity and responsibility to access and assess the researchers’ reasons for excluding certain groups. These reasons should be clear and open to assessment by fair‐minded experts of the field through reasoning and deliberation. Here are examples of questions that enforcers should be able to consider:

- Have the researchers adequately contextualized and justified the exclusion of specific databases or datasets from their analysis?
- Do the reasons provided by the researchers for exclusion align with the study's objectives and the potential societal advantages of the research?
- Do the benefits of excluding specific data (such as enhancing statistical power or mitigating the influence of confounding variables) proportionally outweigh the drawbacks (such as potential underrepresentation of a particular group and limited applicability of findings to its members)?

Certainly, enforcement should extend beyond merely penalizing researchers who fail to adequately justify their exclusions. Education about the new requirements and what constitutes relevant or irrelevant reasons will be instrumental. Furthermore, it is essential that the guidelines adopted by funders, IRBs, DACs, and publishers to operationalize the A4R process do not place an undue burden on the stakeholders, such as external reviewers and authors. Expectations should be grounded in the reality and context of the field and communicated to researchers well in advance of the submission stage to avoid any surprises. The research community should play an active role in this transition and should be informed in advance about the implications for the review processes.

To enforce equitable research practices, we put forth several suggestions for implementation across various levels. First, in the realm of funding, we recommend the inclusion of an evaluation criterion centered on methods that ensure equity and inclusivity in research. The Canadian Institutes of Health Research,^17^ Canada's health research funding agency, have already integrated criteria related to representation, sex, and gender in their evaluation process. Expanding these criteria to encompass diversity and include underrepresented populations is a logical step forward. Additionally, for DACs, we propose the incorporation of a dedicated section offering guidance on methods aiming to facilitate equitable and inclusive research, especially concerning data acquisition. This approach will harmonize data access procedures with principles of diversity and inclusivity, thereby contributing to a more comprehensive and just research environment. Within the sphere of IRBs, our focus should be on heightening awareness among those responsible for protocol review about the significance of diversity and inclusion in genetic research, along with the detrimental consequences of nondiverse samples. Over time, these efforts will result in the development of refined guidelines, akin to those established by the Tri‐Council Policy Statement for the ethical conduct of research involving humans,^18^ and will better address the distinctive requirements of genetics research and nurture a culture of diversity and inclusion within the field.

***Revision***. The external review process is the ideal stage to incentivize researchers while allowing ongoing deliberation about what constitutes fair and unfair exclusion in various circumstances. As previously emphasized, the assessment of the reasons given by researchers for excluding participants or datasets from their analysis should be contextually informed. What constitutes fair exclusion at one stage of a study may not be deemed fair a few months later at another stage. Scientific consensus on a particular element relevant to the study may have evolved. Moreover, not everyone involved may have equal awareness of potential methodological or analytical alternatives that could minimize the need for exclusion without imposing significant costs on the research team. Due to these factors and the potential for differing opinions among involved parties regarding the relevance of provided reasons, it is imperative that the evaluation process be constructive and promote deliberation, rather than functioning as a dead end for researchers. Reviewers should also maintain transparency by effectively communicating any concerns they identify regarding the justifications presented by researchers. If one or more justifications are deemed unacceptable or inadequate, reviewers should clearly indicate the available options for researchers to modify their protocol or manuscript to make it acceptable. A4R considerations alone should likely not lead to outright rejection but to a “reconsider pending revisions” type of decision, allowing the authors to consider the reviewers’ critical assessment and address their questions and queries where possible. In other words, authors might make the recommended revisions, or they might make a case that some of the reviewer's suggestions cannot or should not be followed by providing reasonable counter arguments. Reviewers should be able to identify which unexplored alternatives the authors could have considered to avoid harmful exclusion. In most cases, requests should be both relevant and feasible, grounded in justification categories that a fair‐minded person would likely find admissible and adequate, considering the study's specific context. Additionally, these requests should align with the circumstances prevailing during the earlier phases of research.

## Starting Point for Ongoing Refinement

The A4R framework can contribute to fostering equity and accountability in the research process. The use of procedural justice frameworks holds promise in advancing distributive justice by enhancing the consideration of diversity in data selection practices. We contend that scientists possessing in‐depth knowledge and expertise relevant to the subject matter under review, if adequately informed and guided by the concerns, views, and positions of the other stakeholders, are well equipped and positioned to critically evaluate the legitimacy of the technical justifications presented by the authors regarding their choices to incorporate or omit data from minority groups.

We acknowledge that the A4R framework has limitations. First and foremost, its lack of specificity can lead to different interpretations of what constitutes a “relevant reason.”^19^ Uncertainty about relevant reasons is already present in the current system, however, given the absence of a general decision framework. To help with this, the A4R framework could be complemented by a list of predetermined criteria for assessing the relevance of reasons provided by authors to justify their exclusion of data from minority groups. This could take the form of a list of valid versus invalid reasons for exclusion in different contexts, which would be determined and updated by a particular research community—the genomic or artificial intelligence research community, for example—and should be clearly articulated so that both the researchers and reviewers have a shared understanding. Otherwise, there is a risk of unfair manuscript rejection based on arbitrary interpretations by peer reviewers of what constitute acceptable or unacceptable reasons for excluding minority data in their field.

Moreover, real‐world application can be challenging due to factors such as power imbalances and limited resources, which hinder complete transparency and inclusivity in the decision‐making processes. When implementing the framework, researchers and those evaluating their research protocols and manuscripts must not overlook the cultural and socioeconomic contexts that can affect what is considered “reasonable” or “fair” within a particular context. The reasonableness of exclusions may depend on local jurisdictions and communities involved in the research. Lastly, while transparency would help in making reasons visible to all stakeholders, the A4R framework offers limited guidance on how to effectively involve and engage different stakeholders, particularly those who are marginalized or less powerful, which could result in tokenistic participation rather than meaningful involvement.

We believe that the A4R framework could offer a pivotal starting point for scientists to enhance fairness within their research practices. It has the potential to extend beyond genomics to other biological or digital research domains, such as the development of applications based on artificial intelligence models. The framework's limitations notwithstanding, it can stimulate ongoing discussion and refinement in research and ultimately contribute to a more equitable and inclusive landscape for scientific inquiry across diverse fields.

## Authorship Statement

Charles Dupras and Hazar Haidar are co‐lead authors.

## Disclosure

Marie‐Pierre Dubé has received minor equity interest in Dalcor Pharmaceuticals.
